# Reducing Antigenicity and Improving Antioxidant Capacity of β-Lactoglobulin through Covalent Interaction with Six Flavonoids

**DOI:** 10.3390/foods12152913

**Published:** 2023-07-31

**Authors:** Pei Pu, Zhifen Deng, Lang Chen, Han Yang, Guizhao Liang

**Affiliations:** Key Laboratory of Biorheological Science and Technology, Ministry of Education, Bioengineering College, Chongqing University, Chongqing 400044, China

**Keywords:** β-lactoglobulin, flavonoids, conjugation, antigenicity, antioxidant capacity

## Abstract

β-lactoglobulin (β-LG) is a pivotal nutritional and functional protein. However, its application is limited by its antigenicity and susceptibility to oxidation. Here, we explore the impact of covalent modification by six natural compounds on the antigenicity and antioxidant characteristics of β-LG to explore the underlying interaction mechanism. Our findings reveal that the covalent interaction of β-LG and flavonoids reduces the antigenicity of β-LG, with the following inhibition rates: epigallocatechin-3-gallate (EGCG) (57.0%), kaempferol (42.4%), myricetin (33.7%), phloretin (28.6%), naringenin (26.7%), and quercetin (24.3%). Additionally, the β-LG–flavonoid conjugates exhibited superior antioxidant capacity compared to natural β-LG. Our results demonstrate that the significant structural modifications from α-helix to β-sheet induced by flavonoid conjugation elicited distinct variations in the antigenicity and antioxidant activity of β-LG. Therefore, the conjugation of β-LG with flavonoids presents a prospective method to reduce the antigenicity and enhance the antioxidant capacity of β-LG.

## 1. Introduction

Cow’s milk primarily consists of 80% casein and 20% whey protein. The whey protein fraction is composed mainly of β-lactoglobulin (β-LG), α-lactalbumin, and serum albumin [1]. Notably, β-LG is absent in human and rodent milk [2]. As we know, β-LG is a naturally occurring soluble globular protein with two primary genetic variants, namely type A and type B. It is composed of 162 amino acid residues, one thio group, and two disulfide bonds, resulting in a monomeric molecular mass of 18.4 kDa [3,4]. β-LG is a highly valuable protein for its rich nutrition and emulsifying and foaming properties, so it is often used as a functional additive in a variety of industries [5]. However, β-LG is a prominent milk allergen, with approximately 82% of individuals with milk protein allergies experiencing an allergic reaction to β-LG [6]. Furthermore, β-LG exhibits limited antioxidant capacity, which compromises its ability to counteract oxidative species, thereby leading to structural, functional, stability, and nutritional alterations [7]. While β-LG can act as a natural carrier for bioactive substances, its inherent antioxidant capabilities alone are insufficient to protect other ingredients, such as oil, from lipid oxidation. Nonetheless, β-LG facilitates the emulsification and stabilization of different oil-in-water emulsions [8]. Consequently, the practical application of β-LG is considerably restricted.

Flavonoids, a group of compounds, are prevalent in vegetables, fruits, seeds, flowers, and bark. They can be classified into various subclasses, including flavones, flavanones, isoflavones, flavanols, and chalcones [9,10]. At present, flavonoids have demonstrated a range of health benefits, which make them applicable in medicine, food, agriculture, and other fields with expanding applications [11,12,13,14,15].

Flavonoids have been extensively employed to optimize the functional properties of proteins [16,17,18]. During food processing, phenolic hydroxyl groups of flavonoids bind to amino or carboxyl groups of proteins, inducing the production of complexes through noncovalent and covalent interactions [19,20]. However, noncovalent interactions can be influenced by the solution or environment and exhibit reversible phenomena [21,22]. Covalent interactions, on the other hand, involve the generation of irreversible covalent bonds between proteins and flavonoids, yielding increased stability, durability, and strength of interaction [16,23]. Recently, investigations on the covalent interaction between proteins and flavonoids have gained significant attention. For instance, Wu et al. obtained covalent conjugates of β-LG with chlorogenic acid and epigallocatechin-3-gallate (EGCG) using a free radical method, which effectively reduced the antigenicity and enhanced the antioxidant performance of β-LG [24]. Wei et al. prepared covalent conjugates of four milk proteins with EGCG through an alkaline method and observed that the covalent conjugates exhibited improved emulsion characteristics compared to noncovalent complexes [25]. In our previous study, we investigated the noncovalent interaction between β-LG and six flavonoids (phloretin, EGCG, naringenin, quercetin, myricetin, and kaempferol, Figure S1) to reduce its antigenicity, elucidating their binding patterns [26]. However, the effects of covalent interactions on the structural and functional characteristics of β-LG still remain unclear.

Here, we examined the covalent modification of β-LG by six flavonoids to assess its impact on the structural characteristics related to the antigenicity and antioxidant performance of β-LG. The findings can provide a valuable approach to reduce allergenicity and enhance the antioxidant capability of β-LG, thus expanding its potential applications.

## 2. Materials and Methods

### 2.1. Materials and Chemicals

Bovine β-LG (protein content ≥ 90%), and six compounds (phloretin, EGCG, naringenin, quercetin, kaempferol, and myricetin, HPLC ≥ 95%) were provided by Sigma Chemical Co., Ltd. (Shanghai, China). Reagents like 1,1-diphenyl-2-picrylhydrazyl (DPPH) and 2,2-azinobis(3-ethylbenzthiazoline)-6-sulfonic acid (ABTS) were obtained from Shanghai Macklin Biochemical Co., Ltd. (Shanghai, China). α-cyano-4-hydroxycinnamic acid (CHCA) was produced by Beijing Solarbio Technology Co., Ltd. (Beijing, China). Unless specifically mentioned, the remaining materials and chemicals met the criteria of being of analytical grade or the highest available grade.

### 2.2. Preparation of β-LG-Flavonoid Conjugates

β-LG-flavonoid conjugates were synthesized using the alkaline method [27]. Briefly, 0.25 g of β-LG was dispersed in 50 mL of ultrapure H_2_O and adjusted to pH 9.0. Sodium azide (0.005 wt%) was added to the solution to inhibit the growth of microorganisms with continuous stirring overnight. Six flavonoids with 50 mg each were dissolved in 5 mL of 70% (*v*/*v*) ethanol–water solution and kept volume to 50 mL with ultrapure H_2_O. After mixing the two solutions together, the reaction was initiated under continuous stirring at 25 °C for 24 h, during which the pH was kept at 9.0. The sample was treated at 4 °C for 48 h by a 3500 Da dialysis membrane to eliminate the unreacted flavonoids, and distilled water was replaced every 6 h. Finally, all samples were lyophilized to obtain vaporous solids of β-LG-flavonoid conjugates. For convenience, the prepared samples were named β-LG-kaempferol, β-LG-myricetin, β-LG-phloretin, β-LG-EGCG, β-LG-naringenin, and β-LG-quercetin, while the untreated β-LG was named natural β-LG and the treated β-LG by the alkaline treatment without flavonoids was named control β-LG.

### 2.3. Matrix-Assisted Laser Desorption/Ionization Time-of-Flight Mass Spectrometry (MALDI-TOF MS)

The molecular weights of β-LG and β-LG-flavonoid conjugates were monitored using the MALDI-TOF MS [24]. In brief, 1 μL samples (natural β-LG, control β-LG, β-LG-flavonoid conjugates) and 1 μL α-cyano-4-hydroxycinnamic acid matrix solution were dispensed into a centrifuge tube, respectively. An aliquot (1 μL) of mixtures were dropped on the MALDI target and naturally dried prior to analysis. Each mass spectrum was acquired in positive ion mode using a MALDI-TOF MS 7090 (Shimadzu, Co., Ltd., Tokyo, Japan). Range of spectrum collection: the first-level mass spectrum range was 10,000–100,000 *m*/*z*, and 500 laser shots were accumulated for each spectrum.

### 2.4. Enzyme-Linked Immunosorbent Assays (ELISA)

The potential antigenicity of all samples (natural β-LG, control β-LG, β-LG-flavonoid conjugates) was estimated by sandwich-ELISA assays employing the commercially available bovine β-LG ELISA kit in line with that of our previous method [26]. The performance of the ELISA kit, as described in the instruction manual, is as follows: (1) Detection Range: 6.25 µg/mL to 200 µg/mL. (2) Sensitivity: The minimum detectable concentration is less than 1.0 µg/mL. (3) Specificity: No cross-reactivity with other soluble analogs. (4) Reproducibility: The intra-assay coefficient of variation is less than 9%, and the inter-assay coefficient of variation is less than 11%. The antigenicity inhibition rate (%) was calculated using the following formula:

$$\mathrm{Inhibition}\mathrm{rate}\left(\%\right)=\frac{C_{0}-C}{C_{0}}\times 100\%$$

where C_0_ is the antigen concentration equivalent of untreated β-LG (natural β-LG, μg/mL) and C is the antigen concentration equivalent of treated β-LG (control β-LG and β-LG-flavonoid conjugates, μg/mL).

### 2.5. DPPH and ABTS Radical Scavenging Capacity of β-LG-Flavonoid Conjugates

The DPPH radical scavenging capacity of the natural β-LG, control β-LG, and β-LG-flavonoid conjugates was measured using the previous method [28]. ABTS radical scavenging capacity was evaluated in the earlier report [29]. The corresponding radical scavenging ability was determined by the following calculation formula:

$$Scavenging\;ability\;\left(\%\right)=\frac{A_{c}-A_{s\;}}{A_{c}}\times 100\%$$

where *A_c_* is the blank group and *A_s_* is the absorbance of the test sample.

### 2.6. Intrinsic Fluorescence Spectroscopy Measurements

The fluorescence measurements were carried out following a previous report with slight modifications [29]. An RF-6000 fluorophotometer (Shimadzu, Co., Ltd., Tokyo, Japan) was employed to conduct the fluorescence investigation at 25 °C using a 10 mm quartz rectangular cell. The intrinsic fluorescence of samples (natural β-LG, control β-LG, β-LG-flavonoid conjugates were dissolved in phosphate-buffered saline, pH 7.2) was measured with a fixed β-LG concentration of 0.2 mg/mL after excitation wavelength at 295 nm. Both excitation and emission bandwidths were set at 5.0 nm, with emission spectra being collected in the wavelength range of 300 to 500 nm. To avoid flavonoids interference, the measured fluorescence intensities of β-LG-flavonoid conjugates were corrected by automatically subtracting those of the corresponding flavonoid solutions without β-LG.

### 2.7. Fourier Transform Infrared (FTIR) Spectroscopy Measurements

FTIR spectra for natural β-LG, control β-LG, and β-LG-flavonoid conjugates were collected by a Nicolet iS50 FTIR spectrophotometer at 25 °C (Thermo Fisher Technology Co., Ltd., Waltham, MA, USA) [30]. The specimens were mixed with KBr and then laminated (using a pressure of 100 kg/cm^2^), and KBr was used as a reference. The spectra (shown as T%) were recorded in the range of 400 to 4000 cm^−1^ with a resolution of 4 cm^−1^ and 32 scans. The final spectra were obtained by subtracting the absorbance of KBr alone from that of all samples, followed by baseline correction and normalization based on the protein peak in the range of 1600 to 1700 cm^−1^.

### 2.8. Circular Dichroism (CD) Spectroscopy Measurements

CD measurements were made at 25 °C with a Chirascan V100 spectrophotometer (Applied Photophysics Ltd., Leatherhead, Surrey, UK) equipped with a quartz cuvette with a 1.0 mm optical path [31]. The protein concentration in the samples (natural β-LG, control β-LG, β-LG-flavonoid conjugates were dissolved in PBS, pH 7.2) was 0.2 mg/mL for far-UV CD spectroscopy and 2.0 mg/mL for near-UV CD spectroscopy. All spectra of samples were collected at wavelengths ranging from 190 to 250 nm for far-UV CD spectroscopy and at wavelengths ranging from 250 to 320 nm for near-UV CD spectroscopy under a nitrogen atmosphere. Structure predictions for β-LG with different treatments were obtained according to the CD spectroscopic data (http://dichroweb.cryst.bbk.ac.uk, accessed on 22 June 2022) [32].

### 2.9. Scanning Electron Microscopy (SEM)

Microstructural changes in β-LG samples were observed using a TM4000 SEM (Hitachi Co., Ltd., Tokyo, Japan) at a 5.0 kV accelerating voltage [33]. Digital images were captured at a 5.00 k× magnification, and some representative ones were presented.

### 2.10. Statistical Analysis

All measurements were conducted at least in triplicate, and the results were expressed as means from 3 separate determinations. Statistical comparisons were determined based on a one-way analysis of variance via Microsoft Office Excel version 2019. Main effect differences with a *p* < 0.05 were statistically significant.

## 3. Results and Discussion

### 3.1. Generation of Covalent Bonds between β-LG-Flavonoids

**Figure 1:**
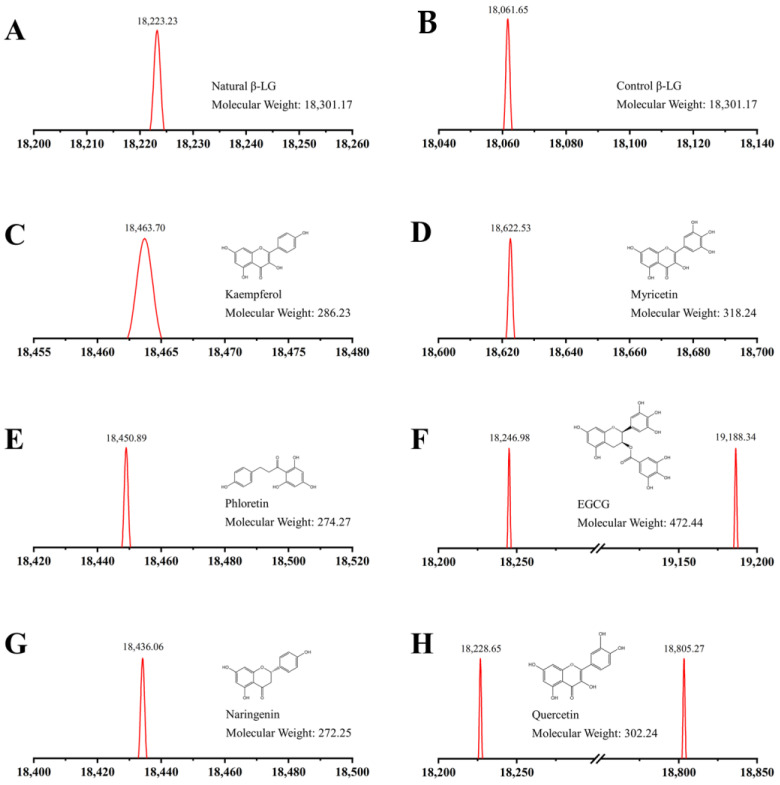
MOLDI-TOF-MS spectra for natural β-LG, control β-LG, and β-LG–flavonoid conjugates. (**A**–**H**) represent the natural β-LG, control β-LG, and the conjugates of β-LG with kaempferol, myricetin, phloretin, EGCG, naringenin and quercetin, respectively.

To confirm the formation of conjugates, the molecular weights of β-LG-flavonoid conjugates were determined using MALDI-TOF MS. In Figure 1, β-LG-kaempferol (18,463.70 Da), β-LG-myricetin (18,622.53 Da), β-LG-phloretin (18,450.89 Da), β-LG-EGCG (19,188.34 Da), β-LG-naringenin (18,436.06 Da), and β-LG-quercetin (18,805.27 Da) exhibited larger molecular weights compared to natural β-LG (18,223.23 Da), indicating covalent bonds between β-LG and six flavonoids were formed.

Previous studies have revealed that the quantity of flavonoids covalently combining with proteins is influenced by the covalent method employed, the structure of the flavonoid, and the solvent environment. For instance, β-LG could covalently bind two molecules of caffeic acid in a pH 2.5 environment and three molecules of caffeic acid in a pH 8.5 environment [8]. The molecular weights of phloretin, EGCG, naringenin, quercetin, myricetin, and kaempferol were reported as 286.23, 318.24, 274.27, 472.44, 272.25, and 302.24 Da, respectively. The discrepancy between naked β-LG and conjugates indicates that one molecule of β-LG can bind one molecule of kaempferol, one molecule of myricetin, one molecule of phloretin, one molecule of naringenin, or two molecules of EGCG or quercetin at pH 9.0. Similarly, Abd El-Maksoud et al. found that one β-LG could covalently bind to one or more polyphenol molecules [34]. This suggests that the covalent binding modes of flavonoids with different structures to β-LG vary.

### 3.2. The Antigenic Characteristics of β-LG-Flavonoid Conjugates

**Figure 2:**
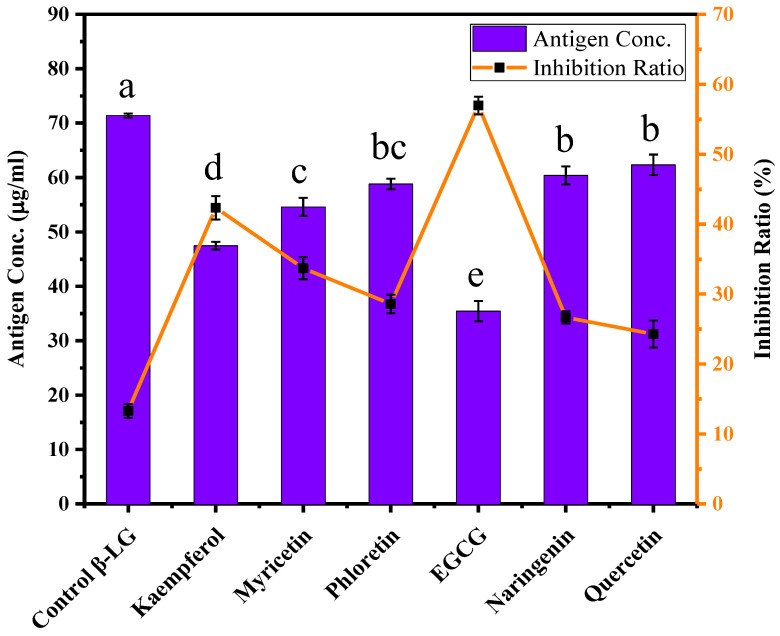
The antigenicity of natural β-LG, control β-LG, and β-LG-flavonoid conjugates. (The a–e represent the significant difference from each other (*p* < 0.05)).

The antigenicity of natural β-LG, control β-LG, and β-LG-flavonoid conjugates was assessed using ELISA. In Figure 2, compared to natural β-LG (equivalent antigen concentration: 82.34 μg/mL), the residual antigen concentration equivalent of control β-LG was 71.39 μg/mL, with a corresponding antigenic inhibition rate of 13.30%. This indicates that alkaline treatment disrupts the conformational epitopes on the protein surface, resulting in decreased antigenicity.

Moreover, Figure 2 demonstrates that the covalent modification of the six compounds led to varying degrees of reduction in β-LG antigenicity. We postulate that flavonoids may covalently alter the conformation of β-LG, resulting in the disruption of its conformational epitopes. Alternatively, the binding sites of flavonoids may be located on or near the antigen epitopes, thereby concealing the epitopes and reducing the antigenicity of β-LG. The β-LG-EGCG conjugate exhibited the lowest antigen concentration equivalent (35.44 μg/mL) and the highest inhibition rate (56.96%). Conversely, the inhibitory effect of quercetin on β-LG antigenicity was the lowest, with an antigen concentration equivalent of 62.34 μg/mL and an inhibition rate of only 24.29%. Interestingly, in our previous study on the noncovalent inhibition of β-LG antigenicity, EGCG also showed more significant inhibitory effects compared to the other five flavonoids, while quercetin exhibited the lowest inhibitory effect [26]. This suggests that both noncovalent and covalent modifications of these two compounds with β-LG share similar preferences or binding poses, resulting in reduced allergenicity of β-LG.

### 3.3. The Antioxidant Performance of β-LG-Flavonoid Conjugates

The antioxidant performance of natural β-LG, control β-LG, and β-LG-flavonoid conjugates was evaluated using DPPH and ABTS radical scavenging assays (Table 1). The DPPH radical scavenging rates of natural and control β-LG were found to be 14.29% and 14.54%, respectively, without significant differences (*p* > 0.05). This indicates that alkaline treatment does not affect the antioxidant capacity of β-LG. However, the DPPH radical scavenging rates of β-LG conjugated with six compounds were increased to varying degrees, indicating that the enhanced DPPH radical scavenging capacity of β-LG-flavonoid conjugates is primarily ascribed to the conjugation of flavonoids. Furthermore, the β-LG-flavonoid conjugates exhibited strong ABTS radical scavenging capacities, with the following ranking: β-LG-phloretin > β-LG-myricetin > β-LG-quercetin > β-LG-kaempferol > β-LG-naringenin > β-LG-EGCG. This enhanced antioxidant performance of β-LG may be associated with two potential pathways for scavenging free radicals, namely, the presence of antioxidant flavonoids on the exterior of β-LG and the exposure of additional antioxidant amino acids resulting from conformational changes in β-LG [7]. It has been proven in many studies that small molecular ligands can enhance the antioxidant potential of β-LG through covalent modification [8,35]. The addition of ferulic acid (FA) significantly improved the radical scavenging activities of DPPH and ABTS of β-LG, reaching 65.06% and 88.22%, respectively [36]. The free scavenging ability of β-LG-flavonoid conjugates is lower compared to β-LG-FA conjugates, possibly due to the tighter structure of β-LG-flavonoid conjugates, resulting in fewer exposed antioxidant amino acid residues [36]. Tao et al. also found that EGCG-modified β-LG possessed great antioxidant activities in terms of scavenging DPPH radicals and chelating ferrous ions, which may stem from the antioxidant capacity of EGCG itself as well as exposure to antioxidant amino acids due to conformational changes in β-LG [37].

### 3.4. The Changes of the Microenvironment around the Tryptophan (Trp) Residue

**Figure 3:**
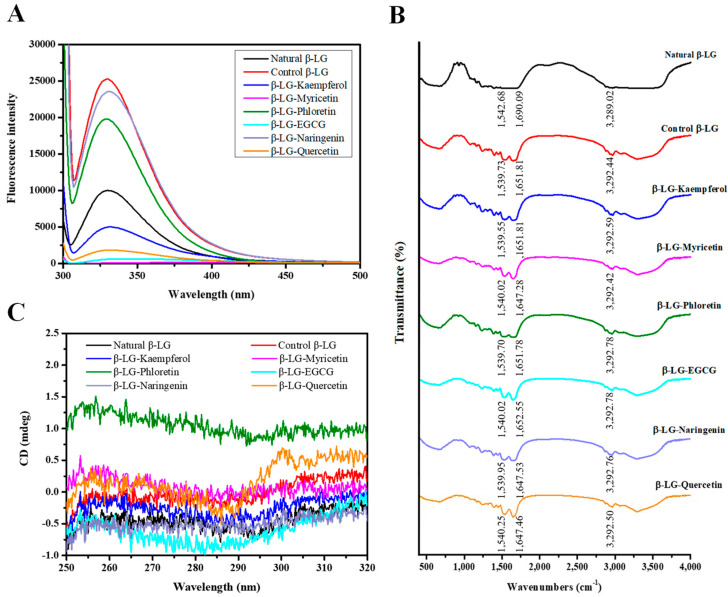
Fluorescence emission spectra (**A**), FTIR spectra (**B**), and the near-UV circular dichroism spectra (**C**) of natural β-LG, control β-LG, and β-LG-flavonoid conjugates.

Selective excitation of the Trp residues in β-LG was performed to probe the microenvironment surrounding them and investigate the effects of flavonoid covalent binding to β-LG on its tertiary structure. Under excitation at 295 nm, the intrinsic fluorescence spectrum (Figure 3A) revealed that both natural and control β-LG exhibited maximum emission wavelengths at 330 nm. However, the fluorescence intensity of control β-LG was significantly higher than that of natural β-LG, indicating that alkaline treatment induced variations in the tertiary structure of proteins, possibly exposing internal fluorophores such as Trp19. The fluorescence intensities of the six β-LG-flavonoid conjugates decreased to varying degrees compared to control β-LG, suggesting that β-LG formed covalent bonds with the six flavonoids and that Trp residues might participate in the covalent reaction with flavonoids, which aligns with the previous finding [38].

The maximum emission wavelengths were found to be 330 nm for β-LG-phloretin, β-LG-naringenin, and β-LG-quercetin, while β-LG-kaempferol, β-LG-myricetin, and β-LG-EGCG exhibited maximum emission wavelengths at 332, 405, and 347 nm, respectively. The observed red shift clearly indicates that conjugate formation leads to an increased hydrophilicity of the microenvironment surrounding Trp residues, while the involvement of Trp in the covalent reaction results in the unfolding of the structure of conjugates, consistent with the findings of Barry [39]. For β-LG-phloretin, β-LG-naringenin, and β-LG-quercetin, no red shift was observed, but the fluorescence intensity was still quenched compared to control β-LG, possibly because of the movement of charged residues near the fluorophore and changes in hydrophilicity. Moreover, the fluorescence intensities of β-LG-myricetin, β-LG-EGCG, and β-LG-quercetin significantly diminished to 181.29, 632.72, and 1855.08, respectively, indicating pronounced shielding effects. This demonstrates that the covalent coupling of myricetin, EGCG, and quercetin to β-LG had a greater impact on its tertiary structure.

### 3.5. The Covalent Interaction of Flavonoids-β-LG

FTIR spectroscopy was employed to further explore the structural changes in β-LG caused by covalent modification with flavonoids. In general, protein structural changes are often manifested through characteristic peak shifts and alterations in peak shape. As depicted in Figure 3B, natural β-LG exhibited three prominent FTIR peaks: 3289.02 cm^−1^ in the amide A band, primarily attributed to N–H stretching and intramolecular hydrogen bonding; 1690.09 cm^−1^ in the amide I band, mainly related to C=O stretching; and 1542.68 cm^−1^ in the amide II band, predominantly reflecting changes in C–N stretching and N–H bending modes. Control β-LG displayed distinct changes in the position and shape of characteristic peaks compared to natural β-LG, indicative of structural variations resulting from alkaline treatment. Notably, the peak shapes of control β-LG in the amide A, amide I, and amide II bands were sharper, signifying the structural perturbations in β-LG induced by alkaline treatment. Additionally, the amide A band peak shifted from 3289.02 to 3292.44 cm^−1^, while the amide I and amide II bands changed from 1690.09 to 1651.81 cm^−1^ and 1542.68 to 1539.73 cm^−1^, respectively. The substantial blue shift in the peak position of the amide I band indicated significant alterations in the C=O bond of β-LG caused by the alkaline treatment.

Compared to control β-LG, the peak positions of the amide A band in the six β-LG-flavonoid conjugates shifted to varying degrees, suggesting the involvement of -NH_2_ groups in β-LG in the covalent reaction between flavonoids and β-LG. Furthermore, Figure 3B demonstrates that three previously reported peaks (3273, 3365, and 3476 cm^−1^) [40] were not observed in our FTIR spectra of β-LG-flavonoid conjugates, indicating the participation of phenolic hydroxyl groups of flavonoids in the covalent reaction with β-LG.

### 3.6. The Structural Characteristics of β-LG Induced by Covalent Modification via Flavonoids

The tertiary structure variations in β-LG were assessed using near-UV CD spectroscopy. In Figure 3C, a peak valley at 295 nm, associated with Trp residues, was observed in the region of natural β-LG. Covalent modification of β-LG with six compounds resulted in shifts of the minima to 290.8, 297.0, 288.4, 282.2, 293.4, and 286.0 nm, respectively, from the initial 295 nm. Furthermore, the molar ellipticities of the different β-LG-flavonoid conjugates around the minima displayed varying degrees of change. These findings suggest alterations in the microenvironment surrounding Trp residues and the spatial structure of β-LG.

**Figure 4:**
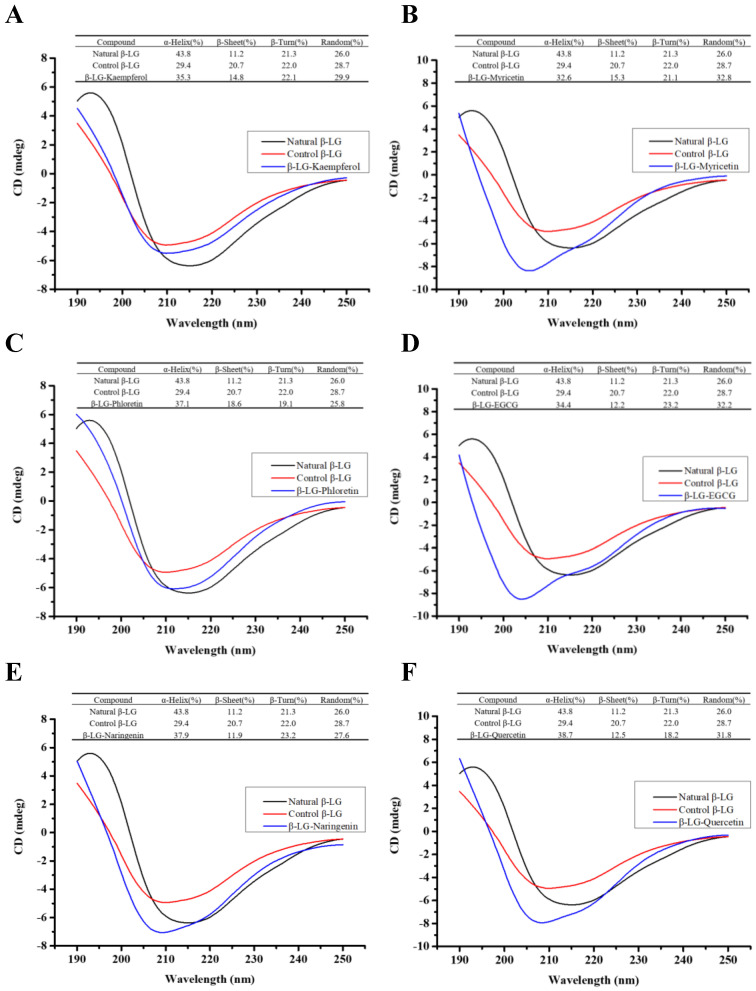
The far-UV circular dichroism spectra of natural β-LG, control β-LG, and β-LG-flavonoid conjugates. ((**A**–**F**) are natural β-LG, control β-LG, and the conjugates of β-LG with kaempferol, myricetin, phloretin, EGCG, naringenin, quercetin, respectively. The insets are the fractions of α-helices, β-sheets, β-turns, and random coils in the absence and presence of flavonoids).

We further investigated the effect of covalent modification with six flavonoids on changes in the secondary structure of β-LG. Figure 4 shows the CD profile of native β-LG, characterized by a negative peak at 216 nm indicating a β-sheet structure, a positive peak at 192 nm, and two negative peaks at 208 and 222 nm corresponding to an α-helical structure. Compared to natural β-LG, the alkaline treatment caused a decline in the α-helical content (from 43.8% to 29.4%), coupled with a rise in β-sheet content (from 11.2% to 20.7%) and random coil content (from 26.0% to 28.7%), indicating that the alkaline treatment induced a more disordered and relaxed protein conformation.

As observed in Figure 4, the α-helical content in β-LG-flavonoid conjugates exhibited significant enhancement compared to control β-LG. Meanwhile, the β-sheet content decreased. Additionally, each conjugate showed distinct changes in random coil content (28.7%), such as an increase in β-LG conjugated with kaempferol (29.9%), myricetin (32.8%), EGCG (32.2%), and quercetin (31.8%), whereas a reduction was observed in β-LG-phloretin (25.8%) and β-LG-naringenin (27.6%). These results indicate a tendency for β-sheet structures in β-LG-flavonoid conjugates to transform into α-helical structures compared to control β-LG. The tendencies of α-helical and β-sheet structures are consistent with those observed in four β-LG-PhA conjugates [34]. The secondary structures of β-LG undergo rearrangement, exhibiting a partially folded state and a more compact conformation. Interestingly, Zhong et al. discovered that the increased β-sheet content and unfolding of the structure resulting from the covalent binding of fructo-oligosaccharides with β-lactoglobulin contribute to the improvement of functional properties and reduction in antigenicity [41].

### 3.7. Morphological Observation of β-LG-Flavonoid Conjugates

**Figure 5:**
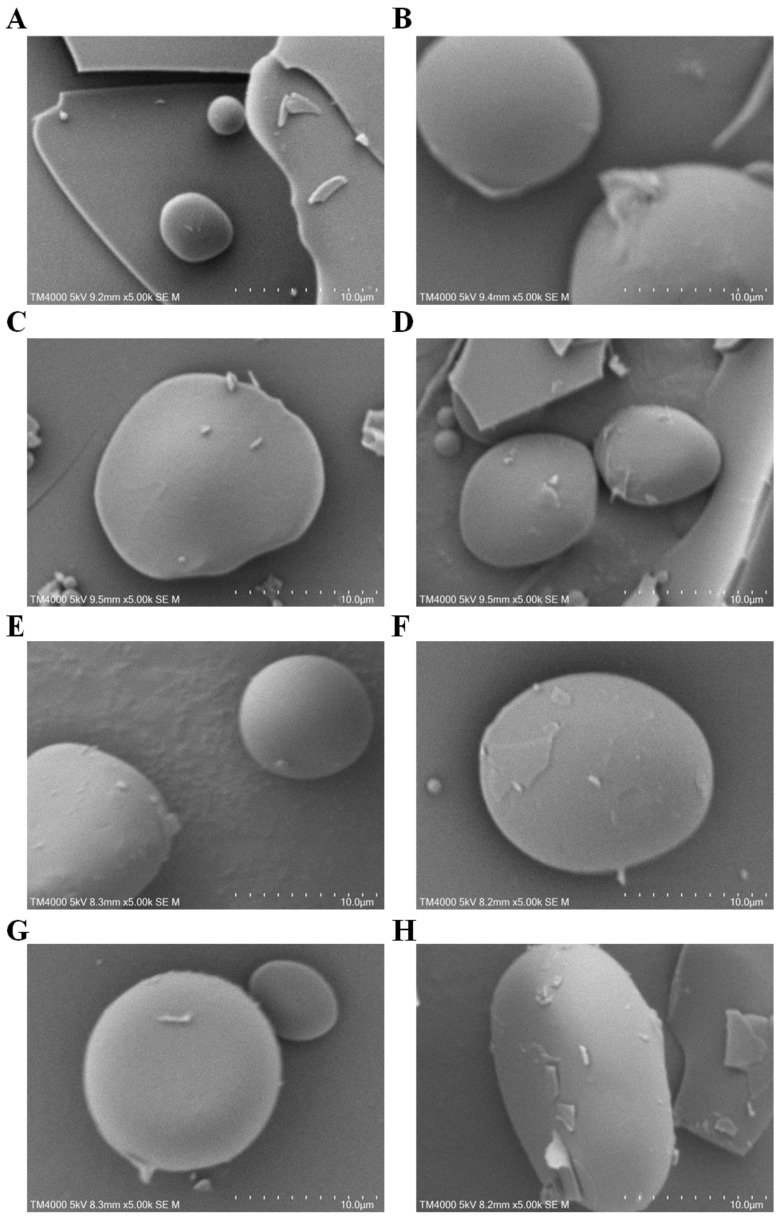
(**A**–**H**) are the scanning electron microscopy of natural β-LG, control β-LG, and the conjugates of β-LG with kaempferol, myricetin, phloretin, EGCG, naringenin, quercetin, respectively.

The morphology of β-LG-flavonoid conjugates was examined using SEM (Figure 5). It is evident that natural β-LG exhibited a compact globular protein morphology with a smooth surface. However, following alkaline treatment, the volume of control β-LG increased while maintaining its spherical structure. This indicates that alkaline treatment can induce alterations in the self-assembly of β-LG molecules, leading to partial unfolding and a looser structure. In comparison, the volume of the six β-LG-flavonoid conjugates showed a slight decrease compared to control β-LG, suggesting that conjugation with the six flavonoids also impacted the self-assembly of β-LG, resulting in a more compact structure. Notably, the conjugates of kaempferol and myricetin with β-LG exhibited irregular spherical structures, whereas the conjugate of quercetin with β-LG displayed an elliptical structure. These findings indicate that the covalent modification based on the three flavonoids (kaempferol, myricetin, and quercetin) exerted a more pronounced influence on the structure of β-LG, aligning with the fluorescence analysis conducted previously (Section 3.4).

## 4. Conclusions

We investigated the impact of six flavonoid compounds on the antigenicity and antioxidant capacity of β-LG and elucidated their mechanism of action by analyzing the structural characteristics of β-LG. We demonstrate that six flavonoids form covalent bonds with β-LG, thereby reducing its antigenicity and enhancing its antioxidant capacity. Notably, EGCG produces the most significant inhibition rate (57.0%) on the antigenicity, and the β-LG-kaempferol conjugate exhibits the strongest DPPH radical scavenging activity (25.70%), while the β-LG-phloretin conjugate presents the greatest ABTS radical scavenging activity (64.43%). The covalent bonds between β-LG and flavonoids involve the -NH_2_ groups of β-LG, resulting in perturbation in the microenvironment around Trp residues and the tertiary structure of β-LG, along with a conversion from α-helix to β-sheet. These structural and conformational changes diminish the immunogenicity of β-LG primarily by disrupting conformational epitopes or masking antigen epitopes and enhance the antioxidant capacity of β-LG, predominantly through the existence of antioxidant flavonoids on its surface or the unmasking of antioxidant amino acids resulting from the structural changes in β-LG.

In the future, the toxicity and digestive characteristics of β-LG-flavonoid conjugates should be investigated using chemical and biomedical experiments. The discovery of numerous natural compounds offers promising avenues for reducing antigenicity or enhancing the antioxidant capacity of β-LG. Bioactive peptides, in addition to small molecular compounds, also hold potential for modifying the antigenicity of β-LG. Our group is currently focusing on identifying antioxidant peptides with the aim of reducing the antigenicity of β-LG, and we plan to publish our findings soon. With the advancement of computer technology, computational methods, particularly artificial intelligence algorithms, provide valuable tools for exploring the interacting mechanisms between compounds and β-LG. Future research will rely on the integration of experimental and computational approaches, which represents an important strategy for investigating the protein-ligand interaction. This study offers valuable theoretical insights and methodological references for reducing or eliminating the antigenicity of β-LG, while also holding significant practical implications for the development of hypoallergenic milk, the enhancement of foods’ nutritional value and processing quality, ensuring food safety, and promoting human health.

## Figures and Tables

**Table 1:** Antioxidant activity of natural β-LG, control β-LG, and β-LG-flavonoid conjugates.

Sample	DPPH Scavenging Activity (%)	ABTS Scavenging Activity (%)
Natural β-LG	14.29 ± 0.04	23.97 ± 0.36
Control β-LG	14.54 ± 0.09	29.04 ± 0.36 ^a^
β-LG-kaempferol	25.70 ± 0.04 ^b,c^	56.07 ± 0.01 ^b,c^
β-LG-myricetin	23.37 ± 0.07 ^b,c^	58.58 ± 0.36 ^b,c^
β-LG-phloretin	16.39 ± 0.04 ^b,c^	64.43 ± 0.05 ^b,c^
β-LG-EGCG	22.29 ± 0.04 ^b,c^	54.04 ± 0.72 ^b,c^
β-LG-naringenin	17.49 ± 0.04 ^b,c^	54.56 ± 0.01 ^b,c^
β-LG-quercetin	22.81 ± 0.07 ^b,c^	56.84 ± 0.03 ^b,c^

^a^ *p* < 0.05 between natural β-LG and control β-LG, ^b^ *p* < 0.05 between natural β-LG and β-LG conjugates, and ^c^ *p* < 0.05 between control β-LG and β-LG conjugates.

## Data Availability

Data is contained within the article.

## Supplementary Materials

The following supporting information can be downloaded at: https://www.mdpi.com/article/10.3390/foods12152913/s1, Figure S1: Chemical structures of six flavonoids (The skeleton structure C6-C3-C6 is labelled as the red (benzopyran: A and C rings) and blue group (phenyl: B ring).
