# Supplementary material for: Reducing Antigenicity and Improving Antioxidant Capacity of β-Lactoglobulin through Covalent Interaction with Six Flavonoids
Source: Foods. 2023 Jul 31;12(15):2913. doi: 10.3390/foods12152913 (PMC10418627; doi:10.3390/foods12152913)
Supplement: Supplementary file 1 [file foods-12-02913-s001.zip › foods-2496468-supplementary.pdf]

Reducing Antigenicity and Improving Antioxidant Capacity of β-Lactoglobulin through Covalent Interaction with Six Flavonoids

Pei Pu †, Zhifen Deng †, Lang Chen, Han Yang and Guizhao Liang *

Key Laboratory of Biorheological Science and Technology, Ministry of Education, Bioengineering College, Chongqing University, Chongqing 400044, China

* Correspondence: gzliang@cqu.edu.cn; Tel.: +86-23-6510-2507

† These authors contributed equally to this work.

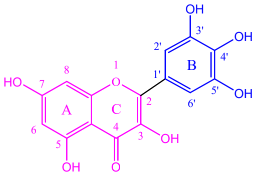

Myricetin

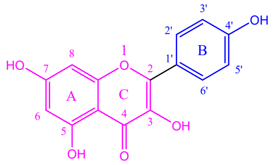

Kaempferol

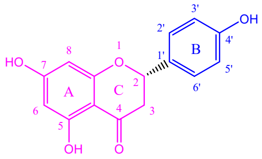

Naringenin

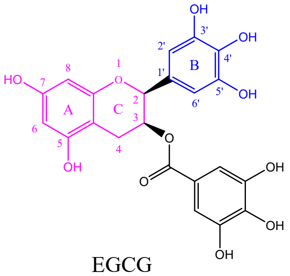

EGCG

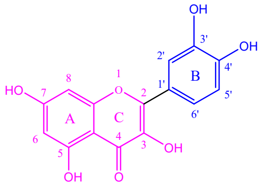

Quercetin

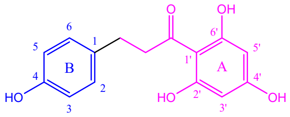

Phloretin

Figure S1. Chemical structures of six flavonoids (The skeleton structure C6-C3-C6 is labelled as the red (benzopyran: A and C rings) and blue group (phenyl: B ring).
